# Use of social media to supplement orthopaedic surgery resident education

**DOI:** 10.1016/j.sipas.2022.100131

**Published:** 2022-09-23

**Authors:** Udit Dave, Wendell W. Cole, Michaela A. Stamm, Mary K. Mulcahey, Collaborative Orthopaedic Education Research Group (COERG)

**Affiliations:** a Tulane University School of Medicine, New Orleans, LA70112, USA; b Department of Orthopaedics, Tulane University School of Medicine, New Orleans, LA, USA

**Keywords:** Social media, Residency, YouTube, Orthopaedic surgery, Program directors

## Abstract

**Objective:**

Social media popularity and utilization have increased in recent years. Past studies have shown high usage of video-sharing platforms such as YouTube as a surgery preparation tool for residents. The growth of social media presents opportunities in orthopaedic resident training, marketing, and networking. The purpose of this study was to determine how orthopaedic surgery residency programs are utilizing social media as a component of their educational curricula.

**Methods:**

An anonymous survey was distributed to program directors for Accreditation Council for Graduate Medical Education (ACGME)-accredited orthopaedic surgery residency programs who are also a part of the Collaborative Orthopaedic Education Research Group (COERG). Program directors completed the survey and forwarded a resident-specific link to residents in their program. Data was collected between September and October 2021. Descriptive statistics were analysed.

**Results:**

A total of 9 program directors (5 M, 4 did not report gender) and 71 (53 M, 8 F, 2 non-binary/gender non-conforming, 8 did not report gender) orthopaedic surgery residents participated in this survey with a majority of participants from the Northeastern United States (3 of 5 program directors, 60.0%; 42 of 64 residents, 65.6%). Residents identified YouTube (24.8%), Instagram (20.0%), and podcasts (20.0%) as the most popular social media platforms. Four of 8 (50.0%) program directors felt that social media use improved their residents’ preparedness for cases, while 3 of 8 (37.5%) felt that it improved teaching of residents. Concerns reported with social media incorporation included confidentiality issues and costs of developing and maintaining social media programs.

**Conclusion:**

Differences exist in the perceived benefits of social media use between orthopaedic surgery residents and program directors. While both groups felt that incorporating social media into training improved case preparedness, only residents felt that their surgical outcomes were improved due to social media use. Most residents viewed the importance of social media incorporation into their training as neutral. This study can serve as a pilot for future studies regarding social media use in orthopaedic surgery resident training due to the improved insight it provides into which social media are being utilized by residents and programs and the attitudes held towards these media.

## Introduction

In recent years, social media has become increasingly ubiquitous, and the incorporation of new technology presents a frontier for innovation [1], [2]. The growing popularity of social media and its high accessibility present an opportunity for inclusion in orthopaedic resident training [2]. Previous studies have identified video-based content, especially YouTube videos, as a highly utilized surgery preparation tool amongst trainees [3], [4], [5]. Unique marketing and networking opportunities are also provided by social networks including Instagram, Facebook, Twitter, Snapchat, and LinkedIn [5], [6], [7]. In addition, social media augments the dissemination of information to both colleagues and patients [8]. It also serves as a valuable tool in orthopaedic resident education given recent challenges and disruptions to in-person medical training posed by the Coronavirus disease 2019 (COVID-19) pandemic [9]. The COVID-19 pandemic forced the majority of didactic teaching to occur in a virtual setting. This resulted in the restructuring of orthopaedic residency protocols altogether which led to an increased reliance on social media to bring together individuals for teaching opportunities [9]. This study was performed after the beginning of the COVID-19 pandemic and therefore reflects social media incorporation and attitudes after major COVID-related changes to orthopaedic surgery education.

There are drawbacks to the use of social media as a tool in surgical training. These include verifying the accuracy of information, preserving confidentiality with regards to Health Insurance Portability and Accountability Act of 1996 (HIPAA) regulations, and a current lack of a robust peer review system [5,6,10]. Although research about the benefits, risks, and utilization of social media in surgical training has been conducted, little is known about the specific usage of and attitudes toward social media by orthopaedic residents and program directors to supplement education in orthopaedic surgery residency programs. Previous studies have shown that social media empowers orthopaedic surgery residents to connect and collaborate with each other effectively and aids in the instruction of orthopaedic surgery residents through teaching and case discussion [11]. To our knowledge, no previous studies have investigated how social media is being utilized as a part of orthopaedic surgery resident education. The purpose of this study was to determine how orthopaedic surgery residency programs are utilizing social media as a component of their educational curriculums.

## Materials and methods

### Study design

An anonymous survey design was used to measure attitudes toward social media amongst orthopaedic surgery residents and program directors. Given that attitudes are not directly observable, we felt that an anonymous survey was the most appropriate design for this study [12]. Study design was evaluated using the Checklist for Reporting of Survey Studies (CROSS) checklist [13], which is included in the appendix.

### Study participants

Study participants included orthopaedic surgery residency program directors (PDs) and orthopaedic surgery residents at Accreditation Council for Graduate Medical Education (ACGME)-accredited residency programs that belong to the Collaborative of Orthopaedic Education Research Group (COERG).

### Survey instruments

An anonymous survey was developed using Qualtrics (Provo, UT, USA).

### Survey dissemination

The anonymous survey was distributed via email to nine orthopaedic surgery residency program directors (PDs). PDs were contacted via email and asked to complete the 20-question Qualtrics survey. PDs were also asked to forward a resident-specific survey link and consent information to their current orthopaedic residents, who received an 18-question Qualtrics survey. The surveys aimed to assess the use of social media in orthopaedic surgery departments in the United States.

### Data collection

Anonymous survey responses and demographic data (i.e., age, sex, home institution, and race) were entered into a data collection tool and stored in a password-protected database. Data was collected between September and October 2021.

### Data analysis

Descriptive statistics were obtained using SPSS Statistics for Macintosh, Version 28.0 (IBM Corp, Armonk, NY, USA). A full list of questions in the surveys included for both program directors and residents can be seen in the Appendix section.

### Ethical considerations

All survey data is confidential and stored in a password-protected database that only the authors have access to. While participants will not experience any direct benefits in participating beside advancing research, there are essentially no ethical issues to consider in this study.

## Results

A total of 9 program directors participated in this survey; however, only 5 of the program directors answered a majority of the survey questions. Our survey reached a total of 120 potential resident respondents, of which 71 residents responded (59.2%). All 5 program directors self-identified as white and 4 of 5 PDs (80%) self-identified as men. Sixteen of the 71 residents (25%) were in their first year of residency and 3 of 5 (60.0%) program directors had been serving at their respective institutions for between 10 and 14 years. Most participating residents (42 of 64, 65.6%) and program directors (3 of 5; 60%) were in institutions in the Northeastern U.S. (Tables 1 and 2). Most participating residents were under the age of 35 years old (63 of 64; 98.4%).

**Table 1:** Demographics of orthopaedic surgery resident participants.

**Demographic Characteristic**	**N (%)**
**Year of Orthopaedic Residency Training** PGY-1 PGY-2 PGY-3 PGY-4 PGY-5	Total: 65 (100%) 16 (24.6%) 13 (20.0%) 13 (20.0%) 11 (16.9%) 12 (18.5%)
**Age (years)** <26 26–30 31–35 36–40 41–45 46–50 >50	Total: 64 (100%) 2 (3.1%) 42 (65.6%) 19 (29.7%) 1 (1.6%) 0 (0%) 0 (0%) 0 (0%)
**Gender** Male Female Transgender Male Transgender Female Non-binary/Gender Non-Conforming Other (please specify) Prefer Not to Say	Total: 65 (100%) 53 (81.5%) 8 (12.3%) 0 (0%) 0 (0%) 2 (3.1%) 0 (0%) 2 (3.1%)
**Race** White/Caucasian East Asian/Pacific Islander/South Asian Hispanic Black/African American Prefer Not to Say	Total: 65 (100%) 48 (73.8%) 5 (7.7%) 4 (6.2%) 3 (4.6%) 5 (7.7%)
**Region** Northeast: (CT, ME, MA, NH, NJ, NY, PA, RI, VT) Midwest: (IL, IN, IA, KS, MI, MN, MO, NE, ND, OH, SD, WI) South: (AL, AR, DE, (D.C.), FL, GA, KY, LA, MD, MS, NC, OK, SC, TN, TX, VA, WV) West: (AK, AZ, CA, CO, HI, ID, MT, NV, NM, OR, UT, WA, WY)	Total: 64 (100%) 42 (65.6%) 16 (25.0%) 6 (9.4%) 0 (0%)

**Table 2:** Demographics of orthopaedic surgery program director participants.

**Demographic Characteristic**	**N (%)**
**Experience as Program Director (Years)** 0–4 5–9 10–14	Total: 5 (100%) 2 (40.0%) 0 (0%) 3 (60.0%)
**Age (years)** <31 31–35 36–40 41–45 46–50 51–55 56–60 61–65 66–70 >70	Total: 5 (100%) 0 (0%) 0 (0%) 1 (20.0%) 3 (60.0%) 1 (20.0%) 0 (0%) 0 (0%) 0 (0%) 0 (0%) 0 (0%)
**Gender** Male Female Transgender Male Transgender Female Non-binary/Gender Non-Conforming Other (please specify) Prefer Not to Say	Total: 5 (100%) 4 (80.0%) 0 (0%) 0 (0%) 0 (0%) 0 (0%) 0 (0%) 1 (20.0%)
**Race** White/Caucasian Black/African American East Asian/Pacific Islander/South Asian Hispanic Prefer Not to Say	Total: 5 (100%) 5 (100%) 0 (0%) 0 (0%) 0 (0%) 0 (0%)
**Region** Northeast: (CT, ME, MA, NH, NJ, NY, PA, RI, VT) Midwest: (IL, IN, IA, KS, MI, MN, MO, NE, ND, OH, SD, WI) South: (AL, AR, DE, (D.C.), FL, GA, KY, LA, MD, MS, NC, OK, SC, TN, TX, VA, WV) West: (AK, AZ, CA, CO, HI, ID, MT, NV, NM, OR, UT, WA, WY)	Total: 5 (100%) 3 (60.0%) 1 (20.0%) 1 (20.0%) 0 (0%)

**Fig. 1:**
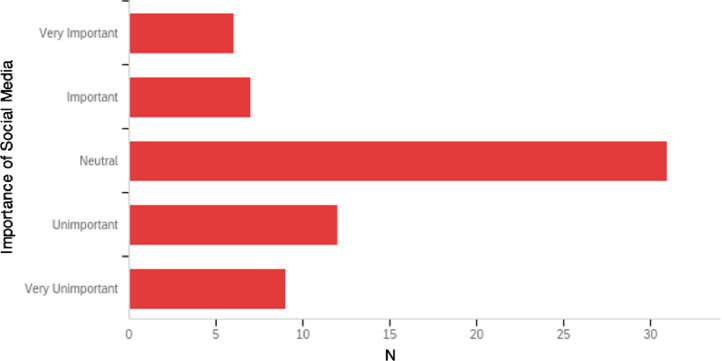
Social media sources utilized by orthopaedic surgery residents to supplement training. Other video sharing and social media sites reported by residents include Vumedi, Orthobullets, and Spotify Podcasts.

Fifty-nine of 65 (90.8%) orthopaedic surgery residents and 5 of 5 (100%) PDs reported that their institutions did not require residents to formally utilize social media. Those residents who reported formal utilization of social media as part of their curriculum cited Instagram, Facebook, and YouTube as the most commonly used social media platforms. Only 17 of 65 (26.2%) residents and 3 of 6 (50.0%) PDs stated that their orthopaedic residency programs encouraged residents to utilize social media. Program directors specified YouTube and OrthoClips podcasts as the media they most often recommend to their residents. A wide range of social media was utilized by orthopaedic residents as an informal supplement to their residency education (Fig. 1). The most popular social media platforms amongst residents included YouTube (24.8%), Instagram (20.0%), and podcasts (20.0%). Social media platforms with the lowest usage by orthopaedic surgery residents included Twitter (4.8%), Facebook (2.9%), LinkedIn (2.9%), Reddit (2.9%), Blogs (1.9%), TikTok (1.0%), Snapchat (0%), and Tumblr (0%), with each source accruing 5% or less resident usage. 12.4% of orthopaedic residents reported using speciality-specific websites to supplement their training.

Both orthopaedic surgery residency program directors and residents perceive tangible benefits from incorporating social media into orthopaedic resident education. Four of 8 (50.0%) PDs felt that social media use improved their residents’ preparedness for cases, while 3 (37.5%) felt that social media use improved the teaching of residents and students. It is noteworthy, however, that none of the orthopaedic residency PDs felt that social media was directly responsible for improving their residents’ surgical outcomes achieved by residents. Additionally, one program director claimed that no educational benefits are associated with social media use and that these tools are used exclusively for improving resident recruitment for the program. Nineteen of 78 (24.4%) orthopaedic surgery residents felt that there are no benefits to incorporating social media into their training. On the other hand, 23 of 78 (29.5%) residents felt better prepared for cases due to social media use, and 16 (20.5%) reported increased confidence with surgical procedures due to the use of social media. Eight of 78 (10.3%) residents felt that their use of social media was directly responsible for them producing positive surgical outcomes in orthopaedic procedures. Ten of 78 (12.8%) residents claimed social media use has benefits in their training pertaining to managing responsibilities such as organization, management, recruitment, and networking.

**Fig. 2:**
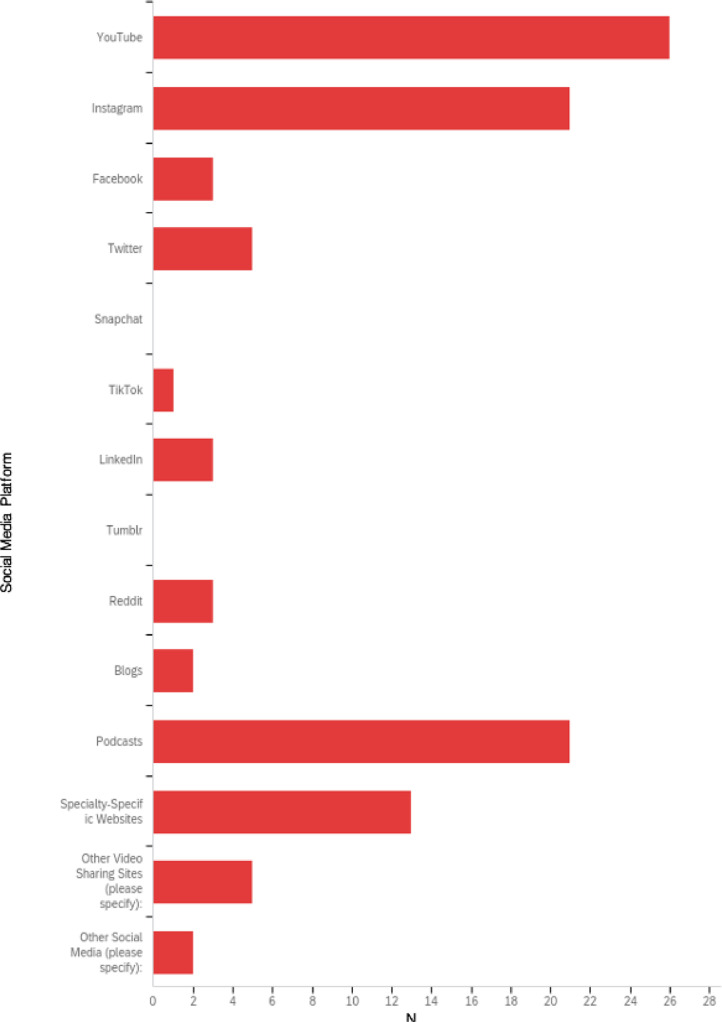
The level of importance placed on social media in orthopaedic surgery training by orthopaedic surgery residents.

All program directors indicated that their orthopaedic surgery programs utilize social media, but 15 of 71 (21%) residents reported that their program does not utilize social media. Seven (46.7%) of these 15 residents suggested that concerns surrounding HIPAA and confidentiality are reasons social media was not used in their programs, while 5 (33.3%) of these 15 residents stated that developing a social media presence would be too time consuming for their respective institutions. Only 4 of 65 (6.2%) residents reported providing feedback to PDs regarding social media use, and 0 of 5 (0%) PDs reported ever receiving social media-related feedback from a resident. Thirty-one of 65 (47.7%) orthopaedic surgery residents viewed the importance of social media as neutral to their training (Fig. 2). Furthermore, 8 of 51 (16%) residents were involved in developing their department's social media presence. However, there was notably no significant difference in the level of importance of social media to orthopaedic residency training ascribed by those involved in the development of their department's social media presence compared to those who were not directly involved (*p* = 0.392).

## Discussion

This study demonstrated that social media has a definite presence in orthopaedic surgery residency programs. The perception of the benefit of social media amongst program directors and residents appeared to differ, as 10.3% of residents believed that social media improved their surgical outcomes, while no program directors reported any perceived connection between resident social media use and subsequent surgical outcomes. Additionally, there may be differences between perceived levels of social media use and feedback. Each program director (5; 100%) reported that their orthopaedic residency program had a presence on social media, but 15 (22.7%) of residents claimed that their program did not use social media in any form. Furthermore, 4 (6.3%) residents reported providing feedback to program directors regarding social media utilization, but 0 program directors reported ever receiving feedback. These discrepancies suggest that the associated programs do not have a robust presence on social media or that they are not actively attempting to involve their orthopaedic surgery residents in social media development and use. This is further supported by the low proportion (8 of 51; 15.7%) of residents who claimed to be involved in the development of their respective orthopaedic residency programs’ social media platforms.

YouTube was found to be the most frequently used social media platform amongst orthopaedic surgery residents. Previous studies reported that most surgical residents use videos to prepare for procedures, with YouTube being the most preferred source [3,5]. Given the high utility of YouTube and other video sharing social media sites for orthopaedic surgery residents, a more overarching, robust peer review system would be beneficial to verify the accuracy of information presented in videos [4]. Large, widely consumed social media sites such as YouTube that present orthopaedic surgery procedure videos as a teaching tool should incorporate a peer review system similar to those employed by Video Journal of Orthopaedics, Orthopaedic Trauma Association, and AO Foundation Surgery Reference, which publish comprehensive peer-reviewed orthopaedic surgical techniques [14], [15], [16].

In addition to the teaching opportunities it provides, social media has the potential to enhance networking opportunities for orthopaedic surgery residents. Since the beginning of the COVID-19 pandemic, there has been a substantial increase in the number of social media accounts for general surgery residency programs [17]. This increase can be attributed to the changes to residency program interviewing structures caused by COVID-19 with a shift to virtual interviewing, which necessitates an increased online presence on the part of programs to best engage with their applicants [17], [18]. In a similar fashion, neurosurgery programs increased their online engagement as a result of the COVID-19 pandemic due to a reduced ability for in person interactions [19], [20], [21]. An increased online presence enhanced the ability of programs to evaluate applicants and applicants to evaluate their fit at programs [19], [20], [21]. Our study demonstrates that social media is a potentially powerful recruiting tool for orthopaedic surgery residency programs as well.

In addition, due to the rapidly evolving nature of social media, orthopaedic surgery programs should increase their awareness of risks associated with social media incorporation (i.e. expensive costs of creating and maintaining programs, hiring managers, patient confidentiality, etc.) [7]. Patient confidentiality was the predominant concern raised by participants of this survey who reported that their institutions did not have a presence on social media.

There are several limitations to this study. First, this was a survey-based study and therefore is subject to the limitations imposed by this study design. Participants were not required to answer all questions; therefore, many participants only chose to answer some questions. This limited the comparisons that could be made because we did not have an equal number of available data points for each question. Further, some questions in the program director survey only had a response from one individual. As most of the responses came from programs and residents in the Northeast, we may not be able to generalize our results nationally.

## Conclusion

Differences exist in the perceived benefits of social media use between orthopaedic surgery residents and program directors. While both groups felt that incorporating social media into training improved case preparedness, only residents felt that their surgical outcomes were improved due to social media use. Most residents viewed the importance of social media incorporation into their training as neutral. This study can serve as a pilot for future studies regarding social media use in orthopaedic surgery resident training due to the improved insight it provides into which social media are being utilized by residents and programs and the attitudes held towards these media.

## Funding

This research did not receive any specific grant from funding agencies in the public, commercial, or not-for-profit sectors.

## Declaration of Competing Interest

The authors declare that they have no known competing financial interests or personal relationships that could have appeared to influence the work reported in this paper.

## Appendix 1 A list of all survey questions presented to both orthopaedic surgery residents and program directors

*Residents*

1. Which year of your orthopaedic residency training are you currently in?

○ PGY-1
○ PGY-2
○ PGY-3
○ PGY-4
○ PGY-5

2. How many faculty members are in your orthopaedic surgery department?

○ 1–5
○ 6–10
○ 11–15
○ 16–20
○ 21–25
○ 26–30
○ >30

3. Does your institution require you to utilize any social media in your orthopaedic resident training (i.e., YouTube videos in curriculum, Instagram/Twitter, etc.)

○ Yes (please specify the most used medium)
○ No

4. How important do you believe social media is for orthopaedic resident training?

○ Very Important
○ Important
○ Neutral
○ Unimportant
○ Very Unimportant

5. Which of the following are ways social media is utilized in your medical training?

○ Preparation for Procedures
○ Marketing
○ Networking
○ Dissemination of Information to Patients
○ Hospital Policy/Training
○ Preparing for the Orthopaedic In-Training Examination
○ Other (please specify)

6. Rank the following uses of social media as they relate to medical training (1 being the most important and 6 being the least important).

○ Preparation for Procedures
○ Marketing
○ Networking
○ Dissemination of Information to Patients
○ Hospital Policy/Training
○ Preparing for the Orthopaedic In-Training Examination or ABOS Part I

7. Does your residency program have an active presence on social media (i.e. YouTube, Instagram, Facebook, etc.)?

○ Yes -> Show 8, 9, 10, 11, 12
○ No -> Show 13

8. Were you involved in the development of your residency program's social media presence?

○ Yes
○ No
○ Not applicable (our program does not have a social media presence)

9. What do you see as the benefits of incorporating social media into your orthopaedic surgery residency education? Please select all that apply.

○ Better preparation for cases
○ Improved surgical outcomes
○ Increased confidence with surgical procedures
○ Improved organization and management of residency responsibilities
○ None
○ Other (please specify)

10. What type of social media do you use to supplement your orthopaedic residency education? Please select all that apply.

○ YouTube
○ Instagram
○ Facebook
○ Twitter
○ Snapchat
○ TikTok
○ LinkedIn
○ Tumblr
○ Reddit
○ Blogs
○ Podcasts
○ speciality-Specific Websites
○ Other Video Sharing Sites (please specify):
○ Other Social Media (please specify):
○ None of the above – I don't use social media to enhance my education

11. Is social media formally incorporated into your orthopaedic training program? If so, how often?

○ Yes
○ Daily
○ Twice a week
○ Once a week
○ Twice a month
○ Once a month
○ Quarterly
○ Yearly
○ Other (please specify)
○ No

12. How much time per week do you spend using social media to supplement your orthopaedic residency education?

○ <30 min
○ 31 min – 1 hour
○ Between 1–2 h
○ Between 2–3 h
○ >3 h
○ N/A – I don't use social media to supplement my residency education

13. Why do you think social media not currently being utilized by your orthopaedic residency program?

○ Too expensive
○ Too time consuming to develop a program
○ Difficulty verifying the accuracy of available information
○ Patient confidentiality/HIPAA concerns
○ Other (please specify)

14. Do you provide feedback to your program director regarding incorporation of social media into your orthopaedic residency education?

○ Yes
○ Anonymously
○ Not anonymously
○ No

15. Which region is your residency program located in?

○ Northeast: CT, ME, MA, NH, NJ, NY, PA, RI, VT
○ Midwest: IL, IN, IA, KS, MI, MN, MO, NE, ND, OH, SD, WI
○ South: AL, AR, DE, (D.C.), FL, GA, KY, LA, MD, MS, NC, OK, SC, TN, TX, VA, WV
○ West: AK, AZ, CA, CO, HI, ID, MT, NV, NM, OR, UT, WA, WY

16. What is your age?

○ <26
○ 26–30
○ 31–35
○ 36–40
○ 41–45
○ 46–50
○ >50

17. What is your gender?

○ Male
○ Female
○ Transgender Male
○ Transgender Female
○ Non-binary/Gender Non-Conforming
○ Other (please specify)
○ Prefer Not to Say

18. What is your race?

○ White/Caucasian
○ Black/African American
○ East Asian/Pacific Islander/South Asian
○ Indigenous American
○ Hispanic
○ Other (please specify)
○ Prefer Not to Say

Program Directors

1. How long have you been the program director of your department of orthopaedic surgery?

○ 0–4 years
○ 5–9 years
○ 10–14 years
○ 15–19 years
○ 20–24 years
○ 25–29 years
○ ≥30 years

2. How many faculty members (including you) are in your orthopaedic surgery department?

○ 1–5
○ 6–10
○ 11–15
○ 16–20
○ 21–25
○ 26–30
○ >30

3. Does your institution formally require residents to utilize any social media in their orthopaedic resident training (e.g., YouTube videos in curriculum, Instagram/Twitter posts, etc.)

○ Yes (please specify the most used medium)
○ No

4. How important do you believe social media is for orthopaedic resident training?

○ Very Important
○ Important
○ Neutral
○ Unimportant
○ Very Unimportant

5. Which of the following are ways social media is utilized in your medical training?

○ Preparation for Procedures
○ Marketing
○ Networking
○ Dissemination of Information to Patients
○ Hospital Policy/Training
○ Preparing for the Orthopaedic In-Training Examination
○ Other (please specify)

6. Rank the following uses of social media as they relate to medical training (1 being the most important and 6 being the least important).

○ Preparation for Procedures
○ Marketing
○ Networking
○ Dissemination of Information to Patients
○ Hospital Policy/Training
○ Preparing Residents for the Orthopaedic In-Training Examination or Board Exam (e.g. ABOS Part I)

7. Does your residency program have an active presence on social media (e.g. YouTube, Instagram, Facebook, etc.)?

○ Yes -> Show 8, 9, 10, 11, 12
○ No -> Show 13

8. Were you involved in the development of your department's social media presence?

○ Yes
○ No

9. What do you see as the benefits of incorporating social media into your orthopaedic surgery education? Please select all that apply.

○ Residents better prepared for cases
○ Improved surgical outcomes
○ Improved teaching of residents and students
○ None
○ Other (please specify)

10. What social media is utilized to enhance resident education in your department? Please select all that apply.

○ YouTube
○ Instagram
○ Facebook
○ Twitter
○ Snapchat
○ TikTok
○ LinkedIn
○ Tumblr
○ Reddit
○ Blogs
○ Podcasts
○ speciality-Specific Websites
○ Other Video Sharing Sites (please specify):
○ Other Social Media (please specify):

11. Is social media formally incorporated into your residency curriculum? If so, how often?

○ Yes
○ Daily
○ Twice a week
○ Once a week
○ Twice a month
○ Once a month
○ Quarterly
○ Yearly
○ Other (please specify)
○ No

12. On average, how much time per week do residents spend using social media to supplement their orthopaedic residency education?

○ 0 min
○ 1–30 min
○ 31 min – 1 hour
○ Between 1–2 h
○ Between 2–3 h
○ I don't know

13. Why do you think social media is not currently being utilized by your orthopaedic residency program?

○ Too expensive
○ Too time consuming to develop a program
○ Difficulty verifying the accuracy of available information
○ Patient confidentiality/HIPAA concerns
○ Other (please specify)

14. Do residents provide feedback to you regarding incorporation of social media into their orthopaedic residency education?

○ Yes
○ No

15. How does your institution measure the impact of social media? Please select all that apply.

○ Survey of residents
○ OITE scores
○ Promotion of participants
○ Increased research output by residents
○ Impact is not measured
○ Other (please specify)

16. Which region is your residency program located in?

○ Northeast: CT, ME, MA, NH, NJ, NY, PA, RI, VT
○ Midwest: IL, IN, IA, KS, MI, MN, MO, NE, ND, OH, SD, WI
○ South: AL, AR, DE, (D.C.), FL, GA, KY, LA, MD, MS, NC, OK, SC, TN, TX, VA, WV
○ West: AK, AZ, CA, CO, HI, ID, MT, NV, NM, OR, UT, WA, WY

17. What is your age?

○ <31
○ 31–35
○ 36–40
○ 41–45
○ 46–50
○ 51–55
○ 56–60
○ 61–65
○ 66–70
○ >70

18. What is your gender?

○ Male
○ Female
○ Transgender Male
○ Transgender Female
○ Non-binary/Gender Non-Conforming
○ Other (please specify)
○ Prefer Not to Say

19. Do you identify as transgender?

○ Yes
○ No
○ Prefer Not to Say

20. What is your race?

○ White/Caucasian
○ Black/African American
○ East Asian/Pacific Islander/South Asian
○ Indigenous American
○ Hispanic
○ Other (please specify)
○ Prefer Not to Say
